# Tocopherol and tocotrienol homologs in parenteral lipid emulsions

**DOI:** 10.1002/ejlt.201400182

**Published:** 2014-08-28

**Authors:** Zhidong Xu, Kevin A Harvey, Thomas M Pavlina, Gary P Zaloga, Rafat A Siddiqui

**Affiliations:** 1 Cellular Biochemistry Laboratory, Methodist Research Institute, Indiana University Health; 2 Department of Medicine, Indiana University School of MedicineIndianapolis, IN 46202, USA; 3 Baxter Healthcare CorporationDeerfield, IL 60015, USA

**Keywords:** HPLC, Lipid emulsions, Tocopherol, Tocotrienol, Vitamin E

## Abstract

Parenteral lipid emulsions, which are made of oils from plant and fish sources, contain different types of tocopherols and tocotrienols (vitamin E homologs). The amount and types of vitamin E homologs in various lipid emulsions vary considerably and are not completely known. The objective of this analysis was to develop a quantitative method to determine levels of all vitamin E homologs in various lipid emulsions. An HPLC system was used to measure vitamin E homologs using a Pinnacle DB Silica normal phase column and an isocratic, n-hexane:1,4 dioxane (98:2) mobile phase. An optimized protocol was used to report vitamin E homolog concentrations in soybean oil-based (Intralipid®, Ivelip®, Lipofundin® N, Liposyn® III, and Liposyn® II), medium- and long-chain fatty acid-based (Lipofundin®, MCT and Structolipid®), olive oil-based (ClinOleic®**),** and fish oil-based (Omegaven®) and mixture of these oils-based (SMOFlipid®, Lipidem®) commercial parenteral lipid emulsions. Total content of all vitamin E homologs varied greatly between different emulsions, ranging from 57.9 to 383.9 µg/mL. Tocopherols (α, β, γ, δ) were the predominant vitamin E homologs for all emulsions, with tocotrienol content < 0.3%. In all of the soybean emulsions, except for Lipofundin® N, the predominant vitamin E homolog was γ-tocopherol, which ranged from 57–156 µg/mL. ClinOleic® predominantly contained α-tocopherol (32 µg/mL), whereas α-tocopherol content in Omegaven® was higher than most of the other lipid emulsions (230 µg/mL).

**Practical applications:**

The information on the types and quantity of vitamin E homologs in various lipid emulsions will be extremely useful to physicians and healthcare personnel in selecting appropriate lipid emulsions that are exclusively used in patients with inadequate gastrointestinal function, including hospitalized and critically ill patients. Some emulsions may require vitamin E supplementation in order to meet minimal human requirements.

## Introduction

Parenteral lipid emulsions are oil-in-water based suspensions made from vegetable and/or fish oils by emulsifying the oils with phospholipids from egg yolk. Lipid emulsions are mostly composed of triglycerides along with a variety of other components that include phospholipids, cholesterol, phytosterols, squalene, and fat soluble vitamins. Lipid emulsions serve primarily as a source of energy and essential fatty acids. However, they are also an important source of vitamin E (tocopherols and tocotrienols) [1–3].

**Figure 1:**
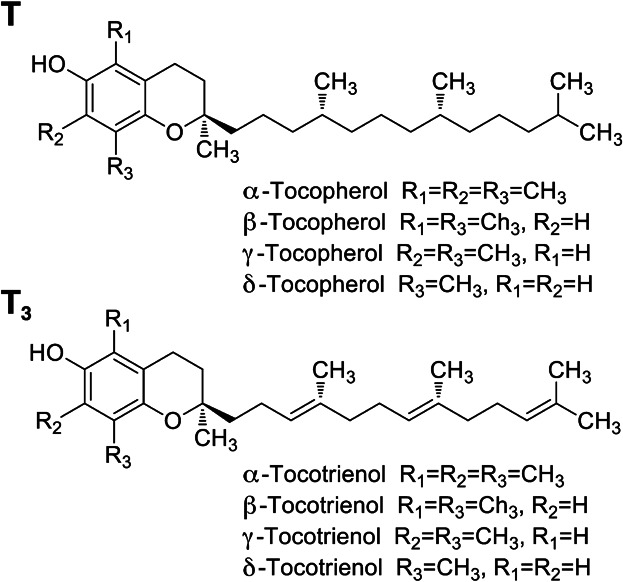
Structure of tocopherols and tocotrienols.All vitamin E homologs (tocopherols and tocotrienols) contain a chromanol ring and a hydrophobic side chain. In tocopherols (T), the side chain is made up of a phytylin group, whereas tocotrienols (T_3_) consist of an isoprenyl group with three double bonds. The T and T_3_ are further classified as either an α, β, γ, or δ homolog, as shown in the figure.

Vitamin E is the generic term for a family of tocopherol and tocotrienol homologs [4–6]. In nature, eight substances have been found to possess vitamin E activity. These substances include α, β, γ, and δ tocopherols and α, β, γ, and δ tocotrienols. All of these compounds feature a chromanol ring with a hydroxyl group that can donate a hydrogen atom to reduce free radicals, and a hydrophobic side chain that allows for penetration of the compounds into biological membranes. Tocotrienols differ from tocopherols by the presence of 3 double bonds on the hydrophobic side chain. ( Figure 1).

Vitamin E homologs have many different biological activities [4, 7]. Vitamin E is the primary fat-soluble antioxidant in the human body [4]. The structure of vitamin E, with its hydrophobic side chain, makes it unique and indispensable in protecting cell membranes from oxidant damage [8, 9]. Due to its preferential accumulation in the body, α-tocopherol arguably is the most important vitamin E homolog believed to modify the course of many oxidative diseases that include cardiovascular disease [10, 11]. In addition to their anti-oxidation properties, several studies have suggested that vitamin E homologs have important effects upon platelets, cholesterol metabolism, and the immune system. For example, tocopherols (γ-tocopherol > α-tocopherol) decrease platelet aggregation and LDL oxidation [12, 13]. Tocotrienols have been shown to inhibit cholesterol synthesis and may reduce the risk of cardiovascular disease [14–17]. α-tocopherol inhibits the activity of protein kinase C (PKC), an enzyme involved in cell proliferation and differentiation in smooth muscle cells, platelets, and monocytes [4, 18]. α-tocopherol has been shown to decrease adherence of blood components to endothelium and modulate enzymes involved in prostaglandin metabolism [4, 19]. Although vitamin E is referred to as the fertility vitamin in animals, its deficiency is not associated with a loss of fertility in humans but has been associated with neuromuscular disease (ie. ataxia, myopathies, neuropathies), immune dysfunction, and anemia [20–22].

Lipid-rich plants and vegetable oils are the main natural sources of vitamin E. However, levels of the various vitamin E homologs differ substantially between oils [23, 24]. α-tocopherol is the main source of vitamin E activity found in supplements and in European diets that are based on olive and sunflower oils, while γ-tocopherol is the most common vitamin E source in the American diet based on soybean, canola, and corn oils. Tocotrienols are high in palm, coconut, and soybean oils. α-tocopherol is the predominant vitamin E homolog in fish oil [25]. However, total vitamin E and α-tocopherol content vary greatly (ie. 0.20–2.25 mg/100g) among different species of fish [25], likely reflecting their different diets. Commercially available parenteral lipid emulsions utilize soybean oil, palm or coconut oil (source of medium-chain triglycerides), olive oil, and/or fish oil. However, levels of vitamin E homologs in these emulsions have not been systematically quantified. The primary objective of this study was to develop a method to quantify the eight vitamin E homologs and to use this method to measure the levels of the homologs in the major commercial lipid emulsions used to treat patients.

Several methods have been used to quantify tocopherols and tocotrienols from oil, bio-fluid and tissue, but the quantification of vitamin E from lipid emulsion had not been reported. In this paper, we report an accurate quantification method for tocopherols and tocotrienols from eleven commercially available lipid emulsions. The method includes liquid-liquid extraction and separation on a normal-phase HPLC with a fluorescence detector. The quantification of the major and minor vitamin E homologs was achieved separately using different external standard curves in the presence of the internal standard.

## Materials and methods

### Materials

Tocopherols (α, β, γ and δ homologs) were purchased from Calbiochem, USA. Tocotrienols (α, β, γ and δ homologs) were purchased from Davos Life Science Pte Ltd, Singapore. *rac*-Tocol was purchased from Matreya LLC, USA. The purity of all reference standards was at least 95%. Hexane (CHROMASOLV®, for HPLC, ≥ 97.0%), methanol (anhydrous, 99.8%), 2, 6-Di-tert-butyl-4-methylphenol (BHT, ≥ 99.0%) and all other reagents were purchased from Sigma-Aldrich (St. Louis, MO, USA). The reference standard and internal standard were dissolved in the hexane solution. All the standard stocks were flushed with N_2_ and stored at −20°C before use.

### Lipid emulsions

Intralipid®_,_ Structolipid®, SMOFlipid®, and Omegaven® were from Fresenius Kabi (Bad Homburg, Germany); Lipofundin® N, Lipofundin® MCT and Lipidem® were from B. Braun (Melsungen, Germany); Liposyn® II and Liposyn® III were from Hospira, Inc. (Lake Forest, IL, USA); Ivelip® and ClinOleic® were from Baxter Healthcare Corporation (Deerfiled, IL, USA). The lipid emulsions are based upon soybean oil, medium-chain triglyceride (MCT) oils,olive oil, fish oil, and a mixture of these oils (Table 1).

**Table 1:** Content of lipid emulsions

Emulsions	Manufacturer	Lot No.	Major component
**Soybean oil-based**			
Intralipid®	Fresenius Kabi (DE)	10BK7082	SO 20 g/100 mL and 1.2 g EYPL
Ivelip®	Baxter Healthcare Corporation (BE)	08K25A92	SO 20 g/100 mL and 1.2 g EYPL
Lipofundin® N	B. Braun (DE)	9173A184	SO 20 g/100 mL and 1.2 g EYPL
Liposyn® III	Hospira, Inc. (US)	70913DW	SO 20 g/100 mL and 1.2 g EYPL
Liposyn® II	Hospira, Inc. (US)	74906DW	Mixture of SO (50%) and SFO (50%), 20 g/100 mL, and 1.2 g EYPL
**Medium- & Long-chain fatty acid-based**			
Lipofundin® MCT	B. Braun (DE)	8494A181	Mixture of SO (50%) and MCT (50%), 20 g/100 mL, and 1.2 g EYPL
Structolipid®	Fresenius Kabi (DE)	10CD2533	Interesterified mixture of equimolar amounts of LCT 64% (w/w) and MCT 36% (w/w), 20 g/100 mL, and 1.2 g EYPL
**Olive oil-based**			
ClinOleic®	Baxter Healthcare Corporation (FR)	09D09A91	Mixture of OO (80%) and SO (20%), 20 g/100 mL, and 1.2 g EYPL
**Fish oil-based**			
SMOFlipid®	Fresenius Kabi (DE)	16CG0134	Mixture of OO (25%), SO (30%), FO (‘5%), and MCT (30%) 20 g/100 mL; and 1.2 g of EYPL
Lipidem®	B. Braun (DE)	9304A181	Mixture of SO (40%), FO (10%), and MCT (50%) 20 g/100 mL; and 1.2 g of EYPL
Omegaven®	Fresenius Kabi (DE)	16CA0022	FO 10 g/100 mL and 1.2 g of EYPL

Abbreviations: BE, Belgium; FR, France; DE, Germany; US, United States; SO, soybean oil; SFO, safflower oil; OO, olive oil; FO, fish oil; MCT, medium-chain triglycerides; LCT, long-chain triglycerides; EYPL, egg yolk phospholipids.

### External standard curve

Stocks of a standard mixture containing all eight vitamin E homologs (200 μg/mL for each compound) were diluted in hexane to generate standard curves for the analysis of the vitamin E homologs. From our preliminary experiments, we realized that it was not possible to utilize a single external standard equation to accurately quantify the vitamin E homologs from lipid emulsions because of extreme variations in the concentrations (0.01 to 300 µg/mL or higher) of various vitamin E homologs in the emulsions. We found that the HPLC fluorescent signals of the test compounds were not completely linear, especially at extremely low concentrations. A single standard curve would potentially overestimate the quantity of a vitamin E homolog that was present in low concentrations. In order to solve this problem, we generated two sets of external standard curves in the presence of different amounts of the internal standard. One set of the standards mixture was for the high vitamin E concentrations, whereas the other set was for the low vitamin E concentrations. For high vitamin E concentrations, a stock solution was diluted in hexane to 50.000, 25.000, 12.500, 6.250, 3.125, 1.563 and 0.781 µg/mL, whereas for low vitamin E concentrations, a stock standard mixture was diluted to 0.781, 0.391, 0.195, 0.098, 0.049, 0.024 and 0.012 µg/mL. The high and low standard dilutions also contained *rac*-Tocol (internal standard) at 5 µg/mL and 1 µg/mL, respectively. A standard curve was generated (triplicate runs) using optimized HPLC conditions as described below, and the concentration of each vitamin E homolog was calculated using an equation Y = aX + b (where Y = concentration of the vitamin E homolog to be determined (µg/mL); X = ratio of peak area of the vitamin E homolog (A_Sample_) to the peak area of corresponding internal standard (A_I-Standard_); and a = slope of the standard curve, b = intercept of the standard curve). The results obtained from both standard curves for high and low concentration are summarized in Table 2.

**Table 2:** External standard equation parameters

	For major components (0.8–50.0 µg/mL)			For minor components (0.01–0.80 µg/mL)		
		
Compounds	a	b	R^2^	a	b	R^2^
α-T	8.5286	0.8572	0.9994	9.6901	−0.0436	0.9528
α-T_3_	8.4591	0.9297	0.9994	9.8504	0.0373	0.9494
β-T	6.3785	0.3098	0.9993	2.4708	0.0147	0.9914
γ-T	6.1502	0.2938	0.9993	2.4220	0.0137	0.9908
β-T_3_	6.4720	0.4399	0.9996	2.6936	0.0138	0.9913
γ-T_3_	6.4717	0.4458	0.9996	2.8990	0.0071	0.9926
δ-T	3.8884	0.2641	0.9996	0.9893	0.0012	0.9996
δ-T_3_	3.7912	0.3001	0.9994	0.9895	−0.00005	0.9997

*Note* Equation: y = ax + b. y: concentration (µg/mL) where a = slope of the standard curve, x = peak area ratio (A_Sample_/A_I-Standard_) and b = intercept of the standard curve.

T: tocopherols; T_3_: tocotrienols.

### Lipid emulsion extraction (for high vitamin E concentration)

200 µL of lipid emulsion (in triplicate) was placed into 10 × 13 mm Pyrex tubes with Teflon-lined screw caps. To these tubes, 40 µL of Internal Standard (IS) (C = 250 µg/mL, in hexane), 800 µL of methanol, and then 2000 µL of hexane (0.05% BHT) were added. Tubes were vortexed for 1 min and then centrifuged at 1400 × g for 20 min to separate the aqueous and non-aqueous layers (2 mL). 200 µL of the top layer (non-aqueous layer) was transferred to the HPLC sample vial for HPLC analysis.

### Lipid emulsion extraction (for low vitamin E concentration)

200 µL of lipid emulsion (in triplicate) was added to the 10 × 13 mm Pyrex tubes with Teflon-lined screw caps. To these tubes, 20 µL of IS (C = 10 µg/mL, in hexane), 800 µL of methanol, and then 2000 µL of hexane (0.01% BHT) were added. Tubes were vortexed for 1 min and then centrifuged at 1400 × g for 20 min to separate aqueous and non-aqueous layers. The entire top layer was transferred to a clean glass tube and dried under N_2_ flow. The residues were dissolved in 200 µL of hexane and then transferred to the HPLC sample vial for analysis.

### HPLC separation and quantification

The HPLC system (SHIMADZU, JP) consisted of a LC-20AT pump, a SIL-20AC auto sampler, and a DGU-20A degasser and was equipped with a RF-10A fluorescence detector and a SPD-M20A diode array detector. The wavelengths of the detector were set at 292 nm for excitation and 330 nm for emission for the identification and quantification of the vitamin E homologs. A Pinnacle DB silica normal phase column (100 × 2.1 mm, 1.9 µm, Restek, USA) was used. The isocratic mobile phase contained 2% of 1, 4-dioxane and 98% of n-hexane, and the flow rate was adjusted to 300 µL/min. Tocopherol and tocotrienol peaks were identified by comparing their retention time to the reference standards. Concentrations of the vitamin E homologs were calculated using the external standard equations as described above.

We also evaluated the accuracy of the vitamin E homolog analysis (% recovery) by spiking the standard mixture (including: α-tocopherol 150 μg/mL, β-tocopherol 50 μg/mL, γ-tocopherol 30 μg/mL, δ-tocopherol 4 μg/mL, α-tocotrienol 30 μg/mL, β-tocotrienol 4 μg/mL, γ-tocotrienol 10 μg/mL and δ-tocotrienol 50 μg/mL) into lipid emulsions. Recovery was calculated by the following equation: R% = (C_se_ -C_e_)/C_s_ × 100, where R (%) is the percent recovery of spiked-in standard; C_se_ is the vitamin E content in spiked emulsion; C_e_ is the vitamin E content in the emulsion; and C_s_ is the content of the vitamin E standard added to the emulsion.

## Results and discussion

It is important to develop analytical methods that allow for quantification of all of the individual homologs of vitamin E. Use of HPLC with both normal phase (NP) and reversed phase (RP) column separation are the most common techniques used for the analysis of tocopherols and tocotrienols [26–28]. To obtain higher sensitivity, various HPLC detectors, including ultraviolet (UV), fluorescence, evaporative light scattering detection (ELSD), and electrochemical detection have been described in the literature for vitamin E analysis. The fluorescence detector appears to be more sensitive and selective than the other detectors [29, 30]. Several studies evaluated protocols for sample preparation, liquid-liquid extraction, and solid phase separation with or without saponification [30, 31]. However, these studies report variable recoveries of different vitamin E homologs. The internal standards are widely used to compensate for the effect of various analytical errors, including sample size fluctuations; however, variations in the recovery of the internal standard and vitamin E homologs during the extraction process strongly affect the quantification. Thus, sample preparation procedures need to be optimized to accurately analyze all vitamin E homologs in a single run.

In a recent study, Amaral et al. (2005) compared the soxhlet extraction and the saponification-extraction method, and found that liquid extraction (solid-liquid or liquid-liquid) provided a comparable recovery of vitamin E homologs. However, the recovery of various vitamin E homologs varied and appeared to be sub-optimal. From these studies, it appeared that the extraction of vitamin E homologs during sample preparation for HPLC analysis was crucial, and, if not performed optimally, could affect the quantification of the compounds. Considering the hydrophobic properties of the vitamin E homologs and the composition of the various lipid emulsions, we used a liquid-liquid extraction protocol employing methanol and hexane to prepare samples for HPLC analysis. The use of the methanol accelerated two-layer separation and also removed the polar component from the sample. We first evaluated the effect of varying methanol amounts (200–1200 μL) with hexane (2000 μL) to extract vitamin E homologs from the standard mixtures (containing *rac*-Tocol). The results are shown in Table 3. Our data indicate that the amount of methanol used in the extraction can affect the quantification of the vitamin E homologs. 800 μL of methanol appeared to be optimal, as less than 800 μL of methanol hindered separation of the methanol and hexane layers, whereas a volume higher than 800 μL resulted in no further improvement in the recovery of the vitamin E homologs. We, therefore, used 800 µL of methanol for the extraction of the vitamin E homologs in our subsequent experiments. As explained in the Methods section, we generated two sets of external standard curves in the presence of different amounts of the internal standard to generate equations for estimating the concentrations. One set of the standards mixture was used for the samples with high vitamin E concentrations, whereas another set was used for the samples with low vitamin E concentrations. The data clearly indicate that this approach resulted in a linear relationship between the detection signal and peak area for different isomers. Furthermore, the limit of detection (LOD) of this method was estimated by determining a concentration that generated a peak five-fold higher than the baseline noise level at the optimized HPLC condition, with an injection volume of 5 µL. The results are listed in Table 4. The data demonstrate that the detection limit was directly related to the vitamin E homolog structure, which was mainly affected by the position of the methyl substitute in the benzene ring.

**Table 3:** Affect of methanol volume on the recovery (%) of tocopherols and tocotrienols^a^^,^^b^

Methanol	200 µL	400 µL	600 µL	800 µL	1000 µL	1200 µL
α-T	104.1	104.5	101.6	105.1	110.8	113.9
α-T_3_	99.7	101.9	99.2	99.6	104.0	104.7
β-T	95.0	95.2	93.3	95.5	100.1	101.2
γ-T	96.6	96.4	94.6	96.4	101.3	102.6
β-T_3_	97.9	105.0	100.2	95.2	101.4	95.9
γ-T_3_	110.6	108.2	105.1	106.6	110.6	109.7
δ-T	100.3	100.3	97.3	99.9	104.1	102.9
δ-T_3_	94.9	94.8	91.6	92.1	91.4	87.1

a Standard mixture used in this experiment: α-T: 150 µg/mL; α-T_3_: 300 µg/mL; β-T: 50 µg/mL; γ-T: 30 µg/mL; β-T_3_: 4 µg/mL; γ-T_3_: 10 µg/mL; δ-T: 4 µg/mL and δ-T_3:_ 50 µg/mL.

b Data shown in the Table is % to the theoretical concentration.

T: tocopherols; T_3_: tocotrienols.

**Table 4:** Method detection limit and recovery

Compounds	LOD (µg/mL)^a^	Recovery (%)^b^
α-T	0.098	100.9 ± 2.4
α-T_3_	0.098	93.8 ± 2.2
β-T	0.012	97.5 ± 3.7
γ-T	0.012	106.6 ± 2.7
β-T_3_	0.012	107.5 ± 2.9
γ-T_3_	0.012	96.1 ± 2.1
δ-T	<0.006	98.1 ± 2.8
δ-T_3_	<0.006	88.8 ± 1.2

a Sensitivity of the method was estimated by determining the lowest limit of detection (LOD) concentration which generated a peak five-fold higher than the baseline noise level (injection volume for HPLC: 5 µL).

b Recovery was evaluated by spiking standards into the lipid emulsions.

T: tocopherols; T_3_: tocotrienols.

The results reported in Table 4 indicate that the procedure for vitamin E homolog analysis resulted in recovery of tocopherols in the 98 ± 4% to 107 ± 3% range and recovery of tocotrienols in 89 ± 1% to 108 ± 3% range.

**Figure 2:**
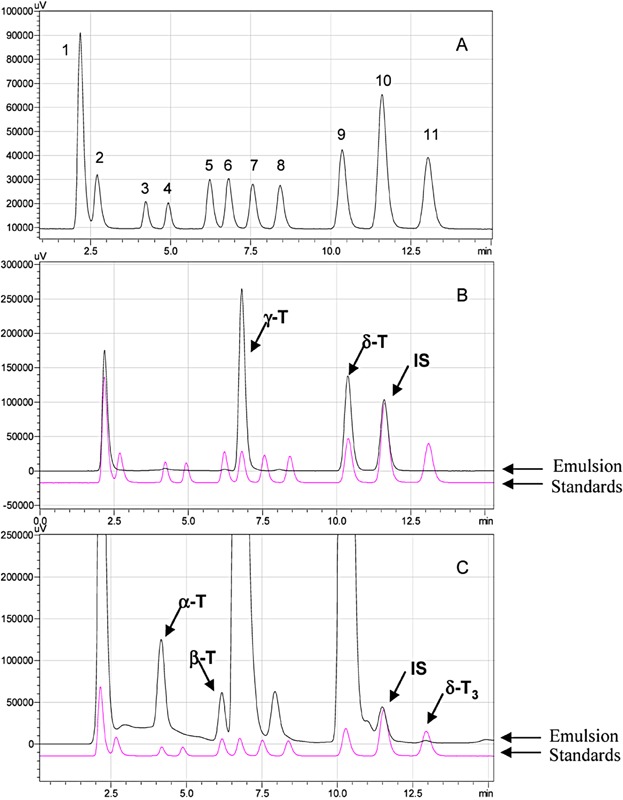
HPLC chromatograph of the reference standard mixture and peak identification for emulsion Liposyn® III.HPLC chromatograph of the reference standard mixture (A): 1: BHT; 2: α-T-acetate; 3: α-T; 4: α-T_3_; 5: β-T; 6: γ-T; 7: β-T_3_; 8: γ-T_3_; 9: δ-T; 10: Internal-Standard (IS, 1.000μg/mL) and 11: δ-T_3_. The concentration of the tocopherols and tocotrienols in the standard mixture are the same (0.391 µg/mL) except α-tocopherol acetate (1.953µg/mL). Peak identification of the major components (B) and the minor components (C) in Liposyn® III were performed using different concentration of a standard solution for a linear response as presented in Table 2 and described in the Method section.

The natural vitamin E contents of the lipid emulsions are significantly affected by the oil species used to manufacture the emulsions. For example, the predominant vitamin E homolog in soybean oil is γ-tocopherol, but in olive and fish oil it is α-tocopherol [32–34]. The content of vitamin E homologs in oils are also further influenced by harvesting, processing, and storage. Thus, the vitamin E homolog composition profile of commercially available lipid emulsions made from different oils by different manufactures may exhibit large variations in vitamin E content [35–40]. The vitamin E contents of the lipid emulsions may also be influenced by the addition of exogenous vitamin E (usually α-tocopherol), which may be added to the lipid emulsions to minimize oxidation of the fatty acids. With the vitamin E quantification method described in this report, we analyzed the tocopherol and tocotrienol content of 11 commercially available lipid emulsions (Table 1 and Table 5). Although different vitamin E homologs have been previously analyzed in different lipid emulsions [35, 40], to our knowledge this is the first study to report the content of all 8 vitamin E homologs in commercial lipid emulsions based upon different oils. The samples were prepared using liquid-liquid extraction protocols for both major and minor components, as described above, and separately run on the HPLC along with corresponding standard mixtures (major or minor component dilutions). An example of a HPLC chromatograph for identifying the major and minor components of tocopherol and tocotrienol homologs is provided for Liposyn® III (soybean oil emulsion) in Figure 2 (A–C). Using this procedure, all the lipid emulsions were analyzed and the data are presented in Table 5.

**Table 5:** Tocopherol and tocotrienol composition in lipid emulsions (µg/mL ± SD)*

	Soybean oil-containing					MCT/LCT		Olive oil- containing	Fish oil-containing		
				
Vitamin E	Intralipid®	Ivelip®	Lipofundin® N	Liposyn® III	Liposyn® II	Lipofundin® MCT	Structolipid®	ClinOleic®	SMOFlipid®	Lipidem®	Omegaven®
α-T	21.02 ± 0.21a	12.86 ± 0.14b	173.05 ± 3.23c	16.18 ± 0.32d	40.37 ± 1.08e	132.02 ± 5.59f	28.35 ± 1.046g	32.03 ± 0.67h	164.50 ± 2.66c	176.72 ± 0.69c	230.12 ± 0.78i
β-T	3.76 ± 0.74a	1.74 ± 0.26b	4.25 ± 0.09c	2.56 ± 0.05d	2.36 ± 0.07d	2.09 ± 0.07d	1.85 ± 0.01b	0.58 ± 0.11e	1.46 ± 0.14f	1.52 ± 0.03f	N/D
γ-T	107.54 ± 0.85a	77.70 ± 0.89b	156.98 ± 1.21c	125.00 ± 0.83d	57.43 ± 0.86e	67.60 ± 1.01f	68.61 ± 0.70f	13.97 ± 0.03g	29.24 ± 0.62h	56.62 ± 0.31e	0.15 ± 0.00i
δ-T	32.99 ± 0.15a	51.22 ± 0.60b	49.56 ± 0.07b	42.27 ± 0.23c	24.52 ± 0.11d	21.21 ± 0.16d	27.73 ± 0.06e	10.51 ± 0.03f	10.71 ± 0.13f	68.82 ± 0.25g	0.01 ± 0.00h
α-T3	N/D	N/D	N/D	N/D	N/D	N/D	N/D	N/D	N/D	N/D	0.67 ± 0.20
β-T3	N/D	N/D	N/D	N/D	N/D	N/D	N/D	N/D	N/D	N/D	0.03 ± 0.00
γ-T3	N/D	N/D	N/D	0.02 ± 0.00	0.09 ± 0.01	N/D	N/D	N/D	N/D	N/D	N/D
δ-T3	0.02 ± 0.00a	0.04 ± 0.00b	0.04 ± 0.00b	0.05 ± 0.00b	0.04 ± 0.00b	0.02 ± 0.00a	0.02 ± 0.00a	0.01 ± 0.00a	0.03 ± 0.04b	0.09 ± 0.00c	N/D
∑T	165.31 ± 1.95a	143.53 ± 1.88b	383.84 ± 4.61c	186.01 ± 1.43d	124.68 ± 2.12e	222.92 ± 6.74f	126.54 ± 1.80e	57.09 ± 0.83g	205.91 ± 3.56h	303.68 ± 1.27i	230.28 ± 0.78j
∑T3	0.02 ± 0.00a	0.04 ± 0.00b	0.04 ± 0.00b	0.07 ± 0.00c	0.13 ± 0.01d	0.02 ± 0.00a	0.02 ± 0.00a	0.01 ± 0.00a	0.03 ± 0.04b	0.09 ± 0.00c	0.70 ± 0.21d
Total	**165.33** **± 1.95a**	**143.86** **± 1.91b**	**383.87** **± 4.61c**	**186.08** **± 1.43d**	**124.81** **± 2.14e**	**222.94** **± 6.74f**	**126.56** **± 1.80e**	**57.93** **± 0.86g**	**205.94** **± 3.59h**	**303.76** **± 1.28i**	**230.98** **± 0.98j**
%T	99.99	99.774	99.99	99.96	99.89	99.99	99.98	98.55	99.99	99.97	99.70
%T3	0.01	0.03	0.01	0.04	0.11	0.01	0.02	0.02	0.01	0.03	0.30

* Data are the average of triplicate samples. N/D, not detected;T: tocopherols; T_3_: tocotrienols; Statistical analyses were done on the normalized data (not adjusted for interdependence) using “R (version 1.15.1)” software (Team RDC (2008) R: A language and environment for statistical computing. Vienna, Austria) from means and standard deviations. Means differences were compared using studentized range with Tukey's hsd (honestly significant difference). Values labeled with dissimilar symbols exhibit significant difference at *p* < 0.05.

Tocopherols (α, β, γ, δ) were the predominant vitamin E homologs for all emulsions, with tocotrienol content < 0.3%. In all of the soybean emulsions, except for Lipofundin® N and Lipofundin® MCT, the predominant vitamin E homolog was γ-tocopherol. In Lipofundin® N and Lipofundin® MCT, the predominant vitamin E homolog was α-tocopherol. The high levels of α-tocopherol in this soybean emulsion are consistent with exogenous supplementation of the emulsion with α-tocopherol. The total vitamin E contents of the soybean oil-based lipid emulsions were highly variable, ranging from 124–384 µg/mL. The vitamin E content of the unsupplemented soybean oil-based emulsions (Intralipid®, Ivelip®, Liposyn® II and III) was less variable and ranged from 124–186 µg/mL. Liposyn® II had the lowest vitamin E content of the soybean oil-based lipid emulsions (mean = 124.81 µg/mL), which reflects its content of both soybean oil and safflower oil.

The content of α-, β-, γ-, δ-tocopherols in different lipid emulsions, as reported in Table 5, is comparable to that of previously reported concentrations for Intralipid® [35], Lipidem® [35], Structolipid® [40] and ClinOleic® [40]; however, we found higher amounts of α- and γ-tocopherols in Lipofundin® N and Lipofundin® MCT compared to that previously reported [35, 40]. The higher α-tocopherol content of Lipofundin® N and Lipofundin® MCT found in the present investigation, as compared to values reported by Wanten et al. (29 μg/mL) [40] and Steger et al. (21.76 ± 2.10 μg/mL) [35], reflects supplementation of the emulsion with exogenous α-tocopherol. The manufacturer likely added exogenous α-tocopherol to the emulsions sometime following the previous studies. The predominant tocopherol of the olive oil-based lipid emulsion, ClinOleic®, was α-tocopherol. This lipid emulsion had the lowest content of vitamin E, which reflects its content in olive oil. Our data for ClinOleic® closely resemble that reported by other investigators [38–40]. It is also important to note that olive oil contains predominantly monounsaturated fatty acids, which are less susceptible to oxidation compared to polyunsaturated fatty acids. Thus, there may have been less evolutionary need for a lipid antioxidant in olive oil. As reported by others [36, 40], our investigation also found α-tocopherol to be the predominant vitamin E homolog in the fish oil-containing emulsions. Despite Omegaven® being a 10% lipid emulsion while the other emulsions were all 20% emulsions, its content of vitamin E (mostly α-tocopherol) was comparable or higher than most of the other lipid emulsions.

## Conclusion

The use of the optimized liquid-liquid extraction procedure and a normal-phase HPLC separation with a fluorescence detector provided high sensitivity and selectivity for the determination of tocopherols and tocotrienols. The two external standard curves, in conjunction with an internal standard, provided an accurate quantification for the tocopherols and tocotrienols in the lipid emulsions. Lipid emulsions contained variable amounts of tocopherols. Tocotrienol content of the lipid emulsions was minimal or not detectable. Additional studies will evaluate the levels of vitamin E homologs in tissues, determine alterations induced by disease, and evaluate the effects of lipid emulsion infusion upon tissue levels.

## Abbreviations

HPLC: high performance liquid chromatography
Vit E: vitamin E
T: tocopherol
T_3_: tocotrienol
